# A Novel Protocol to Assess Acclimation Rate in *Bos taurus* Heifers during Yard Weaning

**DOI:** 10.3390/ani8040051

**Published:** 2018-04-03

**Authors:** Jessica E. Monk, Brad C. Hine, Ian G. Colditz, Caroline Lee

**Affiliations:** 1 CSIRO Agriculture and Food, FD McMaster Laboratory Chiswick, Armidale, NSW 2350, Australia; brad.hine@csiro.au (B.C.H.); ian.colditz@csiro.au (I.G.C.); caroline.lee@csiro.au (C.L.); 2 School of Environmental and Rural Science, University of New England, Armidale, NSW 2350, Australia

**Keywords:** acclimate, cattle, feeding motivation, flight speed, flight time, novelty

## Abstract

**Simple Summary:**

Acclimation protocols for cattle are expected to have benefits for animal handling and production, but the rate at which cattle acclimate has not yet been studied. The current study describes a novel method for assessing the acclimation rate of cattle at a group level during yard weaning. A standardised measure of acclimation rate in cattle will allow further research to be undertaken to identify management practices and selection tools which enhance the capacity of cattle to acclimate to new environments.

**Abstract:**

The speed with which animals acclimate to a new environment could be an important measure of ability to cope with management induced stress. This study developed a measure of acclimation rate in a group of 50 *Bos taurus* heifers during yard weaning over nine days. We recorded the time and order in which heifers moved through a novel funnel structure into a feeding yard daily. We hypothesised that addition of an obstacle at the entrance would increase the time it took heifers to move through the funnel, but that they would acclimate to the obstacle over a three-day period. The change in latency to move through could then be used as a measure of acclimation rate. We hypothesised that individuals which acclimated to obstacles at a faster rate might display favourable temperament as assessed by flight time. All heifers took longer to move through the funnel after a novel object was introduced, then latency decreased over the following two days while the object was present. This indicates the protocol could be useful for measuring acclimation rate at a group level. Individual acclimation rate variables, measured as change in times and orders of heifers between test days, did not appear to have any consistent relationships with flight time or weight change during or post-weaning (*p* > 0.05). We concluded that the protocol was inappropriate for assessing acclimation rate at an individual level, due to social effects while testing heifers as a group. Heifers which were consistently one of the first 20 to move through the funnel had a significantly greater average weight 5 and 10 months post-weaning (345 ± 9 kg and 518 ± 10 kg respectively) than heifers which were consistently one of the last 20 through the funnel (311 ± 8 kg and 484 ± 8 kg respectively; *p* < 0.001). This may indicate order of movement through the funnel was related to feeding motivation or another aspect of temperament not reflected by flight time.

## 1. Introduction

During the course of their lives, livestock are exposed to changes in their environment related to diet, social grouping, management procedures (e.g., weaning, transportation) and human built infrastructure (e.g., animal handling facilities). Responses to these challenges may negatively impact their physiological, behavioural and emotional states and can result in reduced production, poor health and compromised welfare [1,2]. There is therefore interest in the development of methodologies to assess the ability of animals to cope with or acclimate to these challenges. Acclimation is the process by which animals adjust to new environments. Acclimation is thought to arise through a combination of learning processes including habituation, associative learning [3] and physiological adaptation [4]. In accord with this view, previous studies have found that management procedures designed to facilitate acclimation of cattle to human handling improves their temperament, reproductive performance and decreases stress responsiveness to subsequent handling [5].

A number of factors can influence the success of acclimation, such as variation between acclimation methods and length, animal age and prior life experience and variation in the ability of individuals to cope with changing environments. For example, young cattle appear to acclimate to human handling better than mature cattle [5,6]. The reported impact of acclimation protocols on productivity parameters such as average daily weight gain (ADG) has been variable within the literature. For example, Cook et al. [7] reported reduced ADG over 192 days in heifers which underwent an acclimation procedure for 28 days, beginning on day 11 of the trial period. On the other hand, Cook et al. [8] used a similar acclimation protocol but found no impact on ADG over the same time period, which was attributed to differences in level of exercise between protocols. It is important to be aware of factors such as the nature and timing of protocols when developing a method for acclimating cattle to human handling, or for assessing ability to acclimate.

The speed with which individual animals acclimate when experiencing a new environment could be an important and under-utilised measure of ability to cope with handling and the built environment [9]. In a review of the literature on resilience studies in humans, rodents and livestock, Colditz and Hine [10] concluded that assessing responses of livestock during exposure to humans, entry to a new environment (novelty) and change in social structure deserved attention as a method for identifying animals which can better cope with management induced stress. The process of yard weaning [11] in beef cattle provides an ideal time for assessment of acclimation as it is routinely practiced in commercial herds [12], involves each of the challenges noted above and is a sensitive period within the behavioural and social development of animals [13]. We believe a standardised method for quantifying acclimation rate in cattle could be utilised for further research to develop management interventions or to identify and select for animals with a greater capacity to acclimate to new environments.

The objective of this study was to develop a measure of acclimation rate in cattle which could be applied during yard weaning. The method presented in this paper builds on the training procedure described by Walker et al. [11], a conflict-motivation test which required animals to reduce their normal flight distance to move past a stationary human to gain access to a feed reward [14,15]. A similar paradigm was applied in the present study using exposure to novel obstacles of increasing intensity which cattle needed to move past in order to access feed. The key measures in the test were time to move past the obstacle and order in which cattle moved past the obstacle. We propose that a desirable phenotype would be represented by an animal which acclimates faster, as evidenced in this test by a shorter latency to move past the human or other challenging obstacles on consecutive days of testing. We expected this protocol would allow us to measure the acclimation rate of calves at a group level. Additionally, we hypothesised that some cattle in the group would acclimate at a faster rate than others, evidenced by a greater decrease in latency to move past an obstacle on consecutive days of testing relative to other members of the herd. We hypothesised individual acclimation rate would have favourable correlations with flight time, a widely used measure of cattle temperament which is associated with health and production parameters (e.g., [16,17,18,19,20]). Further, we hypothesised individual acclimation rate would have favourable correlations with stress responsiveness and growth.

## 2. Materials and Methods

### 2.1. Animal Ethics

Experimental procedures conducted as part of this experiment were approved by the Commonwealth Scientific and Industrial Research Organisation (CSIRO) McMaster Laboratory Animal Ethics Committee under the New South Wales Animal Research Act 1985 (ARA 16/09).

### 2.2. Animal Details

Fifty heifers (7–9 months old) from the Angus Performance Recording (APR) herd at CSIRO, Armidale NSW were used in this study. The herd had been run on improved pastures across 4 separate mobs, one of which comprised primiparous dams only and the others comprising multiparous dams. Heifers had two prior exposures to humans in the confines of cattle yards, once during marking and again while their dams underwent pregnancy scanning. Heifers were handled by humans in their home paddocks at birth to measure birthweight.

A timeline of procedures conducted during the trial period is given in Table 1. On day 0 of the trial, heifers were weaned from their dams and transported to a second set of yards in a truck (approx. 3.5 km travel distance). Heifers were ear tagged and had numbers branded on their rumps using tail paint for individual identification. Heifers then underwent yard weaning over 10 days. Heifers were weighed on days 0 and 9 of yard weaning then again at 1, 5 and 10 months post-weaning. No mortalities or illnesses were recorded between yard weaning and 10 months post-weaning. During yard weaning, heifers were fed a mix of lucerne hay and lucerne based pelleted concentrate. Lucerne hay was fed at a rate of 1 bale/10 heifers/day. Pelleted concentrate ration was gradually increased during yard weaning, starting from 250 g/heifer/day on day 0 and increasing to 1 kg/heifer/day by day 9.

### 2.3. Haptoglobin Assessment

On days 0 and 3 of yard weaning, 10 mL serum samples were collected from each animal via jugular venepuncture for assessment of haptoglobin concentration, an acute phase protein which can be used as a stress indicator [21]. Blood samples were centrifuged at 2000× *g* for 15 min at 20 °C, then were stored in 2 mL aliquots at −20 °C. Haptoglobin was assessed using a modification of the technique of Jones and Mould [22] as described by Paull et al. [23]. The inter-plate coefficients of variation (CV) for quality control samples containing 0.107, 0.057 and 0.028 mg/mL were 4.14%, 5.93% and 5.88% respectively. Haemoglobin concentrations were also assessed for each sample so that haptoglobin concentration could be corrected for haemoglobin interference as described previously [24]. For the haemoglobin assays, the inter-plate CV for quality control samples containing 1.37, 0.65 and 0.20 mg/mL were 4.28%, 2.68% and 3.24% respectively.

For unknown reasons, 70% of the serum samples had haptoglobin concentrations below the detectable limits of the assay, although standards and quality control samples were in the expected range. After preliminary statistical analyses using the available haptoglobin data, it was determined there were insufficient data to warrant further analyses.

### 2.4. Behavioural Measures

Heifers were Flight Time (FT) tested on days 0, 3, 6 and 9 of yard weaning, then again 1 month post-weaning. Collection of flight time used infrared sensors (Ruddweigh Australia Pty Ltd., Guyra, Australia) to determine the time taken for an animal to traverse a fixed distance of 1.7 m after exiting the crush. Cattle were restrained in the crush for approximately 3 s prior to release for FT measurement. No head bale or lateral squeeze were used during restraint. On day 0, heifers had been weaned and transported prior to FT testing. On all other days, FT testing was conducted prior to, or at least 3 hrs after other procedures.

### 2.5. Acclimation Rate Testing

**Figure 1:**
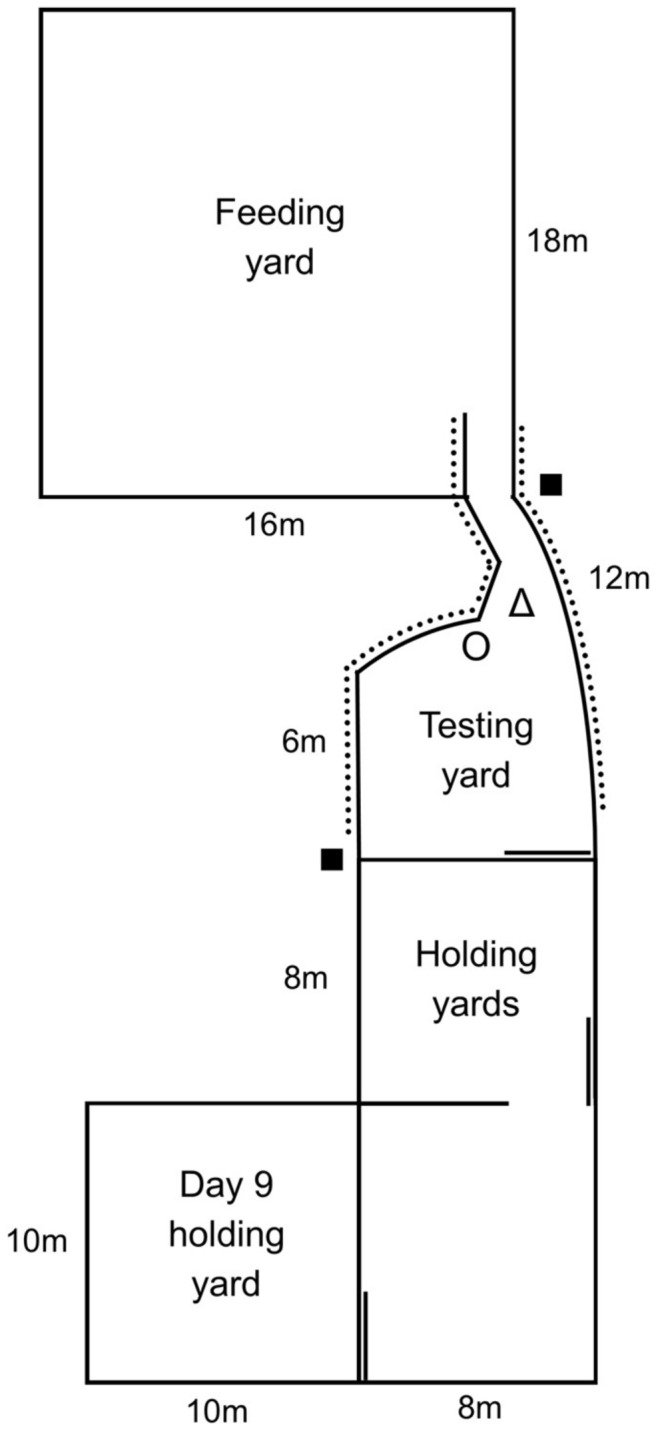
Diagram of the acclimation rate testing set up. The solid square symbols ‘■’ denote the position of two cameras, the open triangle ‘Δ’ denotes the position of a traffic cone in phase 2 of testing and the open circle ‘O’ denotes the position of a person in the final phases of testing. The dotted lines denote which panels in the yards were covered with opaque rubber matting. The figure is approximately to scale.

**Figure 2:**
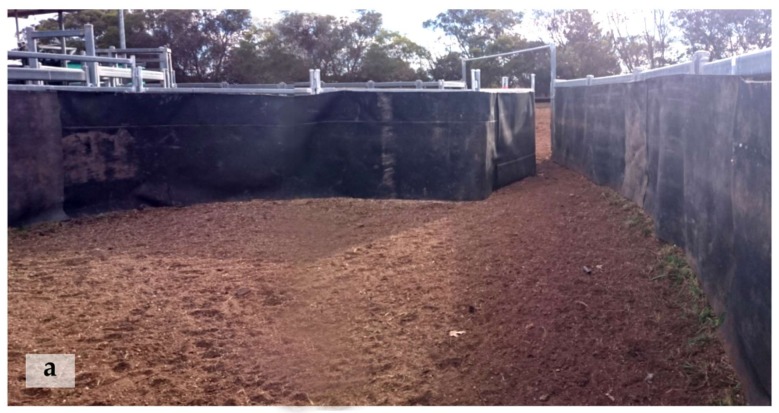

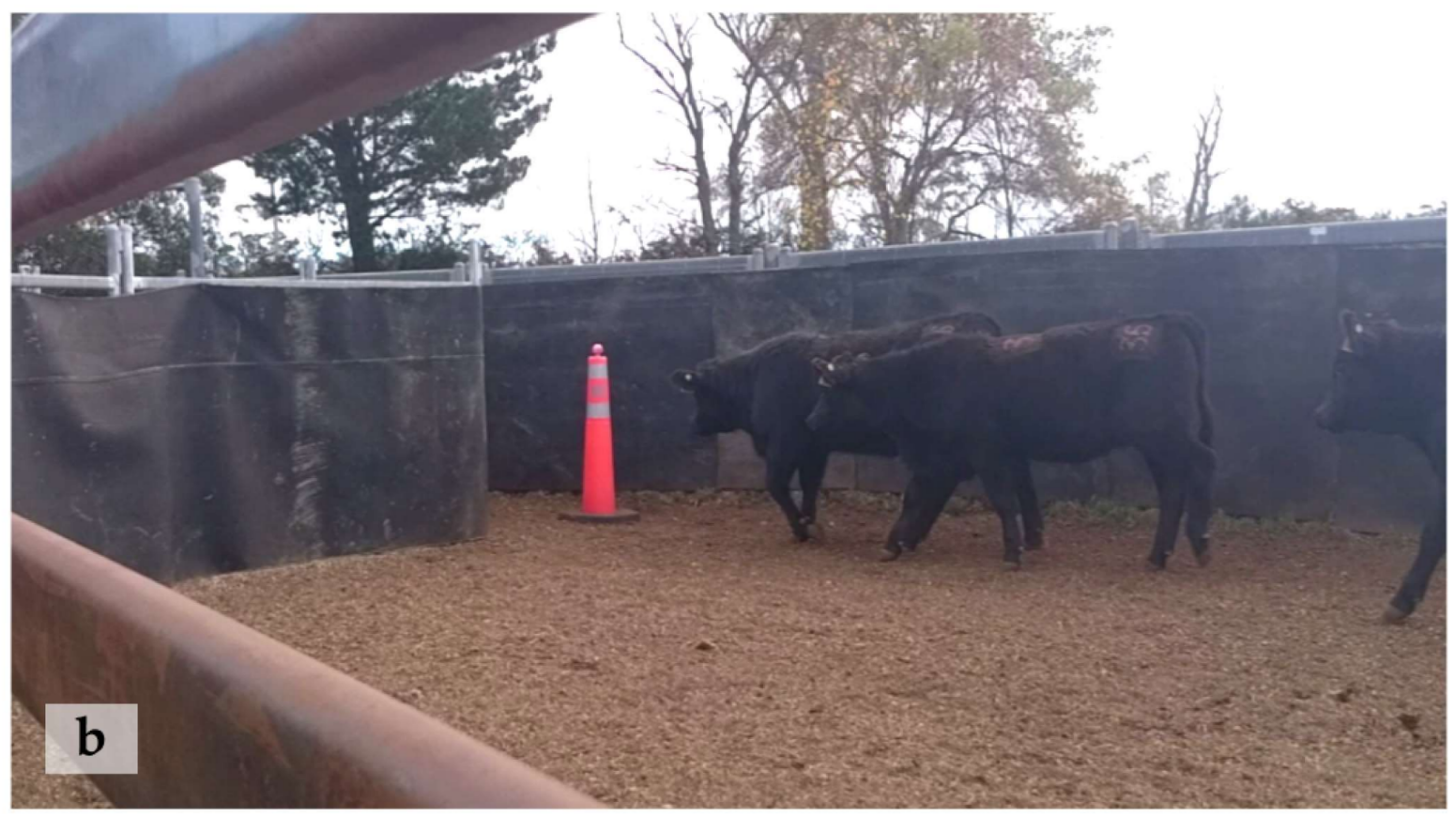
Photographs of the acclimation rate testing set up. Panel (**a**) shows the funnel structure without any obstacle present. Photograph was taken from inside the testing yard looking through to the feeding yard; Panel (**b**) shows the funnel structure on day 4 of testing with the traffic cone in place. This image was taken from a video recording during testing.

To assess acclimation rate, the time and order in which cattle moved through a novel set up to a feeding yard were recorded. The testing setup included 3 areas: the holding, testing and feeding yards (Figure 1). A funnel was constructed in the testing yard with temporary panels so that only one animal could move into the feeding yard at a time (Figure 2). The wall between the testing and feeding yards was lined with opaque rubber matting (Andromeda Industries, Moonbi, Australia). On day 0 of yard weaning, heifers were moved through the set up to the feeding yard by a human handler and left overnight to feed. Acclimation testing then occurred in 4 phases from days 1 to 9 of yard weaning (hereafter referred to as days 1 to 9 of testing). Testing occurred once daily before feeding. All heifers were tested together as a group. If any animals had not moved through the funnel within 10 min, a handler calmly walked into the holding yard and encouraged the remaining cattle through to the feeding yard. Heifers were observed after testing to ensure all animals were eating and had adequate access to food. Animals remained in the feed yard (Figure 1) for a minimum of 2 h after feeding.

On days 1–8 of testing, all heifers were moved into the holding yards, then the gate between the holding and testing yards was opened and a timer started. Heifers could freely move between the 2 holding yards. The times at which each heifer moved through the funnel into the feeding yard were recorded from video footage using a Sony Handycam video camera mounted above the funnel (Figure 1). During the first 3 days of testing (phase 1), no challenges were present in the test. On day 4 (phase 2) a large orange traffic cone, approximately 1.2 m tall, was introduced at the mouth of the funnel (Figure 1 and Figure 2). Heifers had enough space to move around the cone but were forced to walk close to it to enter the feeding yard. The cone remained in this location for days 4–6 of testing. The cone remained standing for the duration of testing on days 4 and 5, but was knocked over part way through testing on day 6. Approximately 15 cattle were left in the holding yard at the time the cone was knocked over. Data after the cone had been knocked over were still used in further analyses.

On days 7 and 8 (phase 3), the cone was removed and a familiar human stood at the mouth of the funnel instead. However due to a number of technical issues prior to and during testing on these days, such as cattle damaging the funnel structure during testing so that they could no longer access the feeding yard, it was determined the data collected were confounded and therefore have not been included in this paper. The protocol was therefore altered for the final day to test heifers individually rather than as a group with a familiar human standing at the mouth of the funnel (phase 4). This allowed for assessment of a different aspect of animal behaviour than the group testing provided. The stationary human at the mouth of the funnel was a familiar female involved in daily care and movement of animals between yards for testing. She stood facing the cattle without making eye contact. All heifers were held in the day 9 holding yard prior to testing, then were moved out by a familiar male human handler one at a time and walked to the testing yard (Figure 1). A timer commenced when the heifer moved into the testing yard and the time it took each animal to move past the human into the funnel was recorded. Day 9 times were ranked for comparison to Order on days 1–6, where the fastest animal was ranked 1 and the slowest animal was ranked 50. We also recorded the order in which they were retrieved from the holding pen by the human handler to be tested, which was largely determined by the animals’ willingness to move past the human.

### 2.6. Statistical Analysis

Data were analysed in R version 3.2.3 [25]. *p* values less than 0.05 were considered significant, values where 0.05 < *p* < 0.1 were considered a tendency towards significance. All model residuals were checked for normality and homoscedasticity using Shapiro–Wilks test for normality and visual assessment of Q–Q and residuals vs. fitted values plots. Day 0 weight was fitted as a covariate in all linear models unless otherwise specified. Dam management group (mob 1–4), calf age at weaning and calf birthweight were fitted as fixed effects in all linear models, however these factors were later removed from all models using a backward elimination approach as none of the factors reached significance in the models. Raw data are provided in the Supplementary Materials.

Changes in mean time to move through the funnel across days 1–6 of acclimation testing were analysed using a maximum likelihood multilevel linear model to account for repeated measures on the same animals across test days [26]. Post hoc analyses were then conducted with the package “multcomp” using Tukey’s contrasts [27].

Spearman’s rank correlation coefficients were calculated to determine consistency of Order of movement through the funnel between pairs of test days. Kendall’s coefficient of concordance (W) was calculated to assess the consistency of Order across groups of test days, specifically days 1–6, 2–6, 1–3 and 4–6. The concordance coefficient ranges from 0–1, where 0 indicates no concordance between days and 1 indicates complete concordance. As suggested by Napolitano et al. [28], we considered W values of <0.4, 0.4–0.6 and >0.6 as low, moderate and high concordance respectively.

Changes in Time to move through the funnel were calculated between adjacent test days and within the test phases (D2–D1, D3–D2, D3–D1 etc.). Pearson’s correlations were then used to assess relationships between the new rate variables and FT, weight, weight change and day 9 test times.

Groups of animals which were consistently first or last through the funnel across multiple days of acclimation testing were identified for further analyses. For each day of testing, we noted whether cattle were one of the first 20 to move through the funnel, one of the last 20 or in the middle of the herd. We then tallied how many times they fitted into each category and assigned them to a Movement Group of “First”, “Last” or “Middle” based on where they were positioned in the group for the majority of the test days. As order on day 1 was not correlated with order on other test days, the Movement Groups were formed using data from days 2–6 only. As such, animals assigned to the “First” group were one of the first 20 animals through the funnel on at least 3 of the 5 test days. Using this classification system, a total of 20 heifers were assigned to the First group, 20 to the Last group and 10 to the Middle group. Due to the relatively smaller number of animals in the Middle group, and because we were primarily interested in the extreme cases, further analyses were conducted using the First and Last groups only.

Differences in mean FT, weight and day 9 test order between the First and Last Movement Groups were analysed using linear models. Differences in mean weight changes between the First and Last Movement Groups were analysed using a maximum likelihood multilevel linear model to account for repeated measures on the same animals across time periods [26], fitting an interaction between Time Period and Movement Group. Post hoc analyses were conducted using the lsmeans package, comparing least-squares means from the model using pairwise contrasts and Tukey P value adjustment [29].

It should be noted that we also determined a movement grouping for the first and last 10 animals to move through the funnel in the same way described previously for the first and last 20 animals to move through the funnel. Preliminary statistical analyses indicated there were not enough animals in the “First 10” and “Last 10” groupings to obtain statistically significant differences between groups and so these data have not been presented.

Pearson’s correlations were used to assess the relationships between time to move through the funnel on day 9, order in which cattle were tested on day 9, FT and weight. Spearman’s rank correlations were used to assess the relationship between order in which cattle were tested on day 9 and with the number assigned to each animal on day 0 of yard weaning, representing their initial order of entry into the yards.

## 3. Results

**Figure 3:**
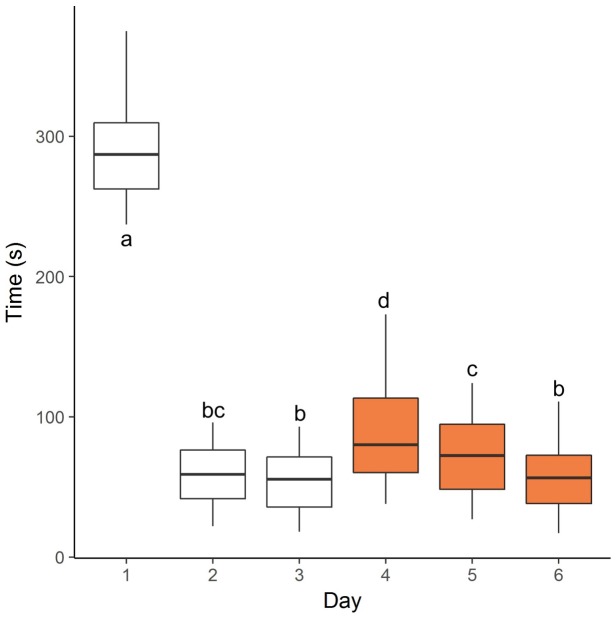
Boxplots showing the minimum and maximum times (whiskers), the first and third quartiles (hinges) and the median times taken for all heifers to move through the funnel structure over consecutive days of acclimation testing (days 1–6). Orange boxes indicate testing days when the traffic cone was present. Three outliers were removed from Day 1 data (timed out at 600 s). Common letters depict least square means which did not differ significantly as assessed using a multilevel linear model.

The mean and median times for all animals to move through the funnel decreased between days 1 and 2, then remained consistent on day 3. Mean and median times increased on day 4 (after introduction of the cone) then decreased again over the following two days (Figure 3).

Order of movement through the funnel on day 1 was not correlated with order of movement on any other test day (*p* > 0.10) (Table 2). Order of movement on days 2–6 were positively correlated with one another, with significant correlations between adjacent test days (Table 2).

Kendell’s coefficients of concordance indicated there was low consistency of Order across test days 1–6 (W_49_ = 0.385, *p* < 0.001), but consistency was improved when day 1 was excluded (W_49_ = 0.478, *p* < 0.001). Consistency during phase 1 of testing was moderate (W_49_ = 0.462, *p* = 0.038) and consistency during phase 2 was high (W_49_ = 0.608, *p* < 0.001).

No correlations were found between acclimation rate variables (changes in Time between test days) and weight, weight change or day 9 test times and orders (*p* > 0.05). Change in Time to move through the funnel during phase 2 of testing (D6–D4 Time) was significantly negatively correlated with day 0 FT (r = −0.32), but not with FT on other days. This means animals which became faster to move through the funnel relative to the rest of the herd on day 6 compared to day 4 were likely to have had a higher FT on day 0. No correlations were found between FT and phase 1 rate variables.

Mean weights of heifers in the First Movement Group were higher than those in the Last group at all time points (Table 3). The differences were not significant for Day 0, Day 9 or 1 month post weaning, but were significant at 5 and 10 months post weaning. No significant differences were found between the First and Last groupings for FT or time to move through the funnel on day 9. Mean order in which cattle were tested on day 9 was notably lower in the First group (22.8) compared to the Last group (30.5), however this difference was not significant (t = −1.6, *df* = 38, *p* = 0.1).

**Figure 4:**
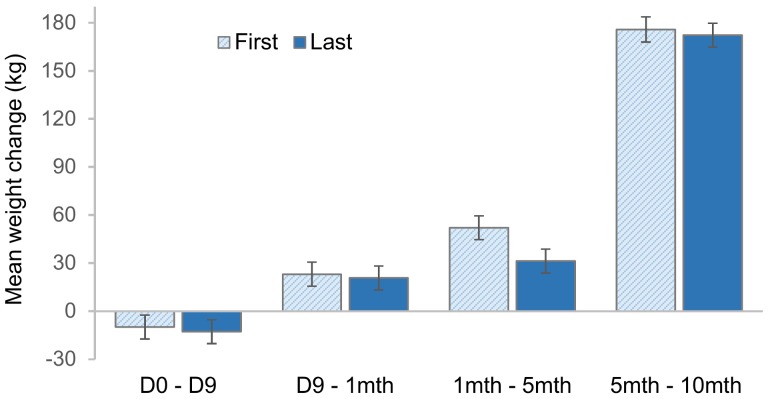
Mean weight change ± 95% CI’s for the First and Last Movement Groups during each time period in the experiment. Heifer weights were recorded at the beginning and end of yard weaning (D0 and D9) then 1, 5 and 10 months post-weaning. Heifers were retrospectively grouped as First or Last depending on whether they were consistently one of the first or last 20 animals to move through the funnel during days 2–6 of acclimation testing. Data were analysed using a multilevel linear model, indicating a significant interaction between Movement Group and Time period (*p* = 0.035). Least-squares means were then used to determine the significance of differences between Movement Groups for each time period.

In the repeated measures analysis on weight change data, the Time Period x Movement Group interaction was significant (F_3,112_ = 3, *p* = 0.035). Time period had a significant effect on weight change (F_3,112_ = 949.8, *p* < 0.001), but Movement Group only tended to have an overall effect (F_1,38_ = 3.1, *p* = 0.085). Post hoc analyses indicated the weight change between months 1 and 5 differed significantly between Movement Groups (t (37) = 3.99, *p* = 0.006) but that differences between groups were not found for any other Time Periods (*p* > 0.10) (Figure 4).

The order in which cattle were tested on day 9 following being moved out by a human handler one at a time was significantly negatively correlated with all FT measures (Table 4). Order of testing on day 9 also tended to be negatively correlated with weight difference during weaning (r = −0.26, *p* = 0.072). The time it took individual heifers to move through the funnel on day 9 did not appear to have any consistent correlations with weight or FT.

## 4. Discussion

The aim of this study was to develop a protocol to assess the rate at which individual cattle acclimate to novel environments, handling by humans and novel objects. In combination, the protocol included three sequential phases of exposure to novel stimuli, together with the totality of husbandry procedures implemented over 10 days of yard weaning, including handling in yards, restraint in a crush/chute, blood sampling, ear tagging and hand feeding. The husbandry procedures employed were typical of commercial practices used during yard weaning. An acclimation assessment protocol implemented at this time would more readily fit into existing commercial practices and allows us to assess how cattle cope with the additional challenges while they are already under stress. As a group, the cohort of heifers in the current study acclimated to the novel objects during the testing procedure, evidenced by a decrease in time to move through or past the obstacles between test days. The decrease in time between days 1 and 2 was likely due to both acclimation to the novel funnel set-up and increased association between the procedure and feed. By day 2, heifers showed little hesitation to move through the funnel with no further decrease in time on day 3. We expect this indicates the association between the protocol and feed had been well established by day 2, which is consistent with previous work during which cattle were trained to move to feed (e.g., [30]). This may mean phase 1 could be shortened to two days in future studies. Introduction of the traffic cone did challenge the heifers, as evidenced by the increase in time to move through the funnel between days 3 and 4. We expect this second phase would be most useful in future for assessing the rate of acclimation in cattle at a group level, focusing on the extent to which the novel object increases movement time initially, then the decrease in time to move through the funnel over the following days. An additional phase with a more challenging obstacle (such as a human) could potentially allow us to consolidate the responses seen during phase 2. Alternatively, and more likely, an additional phase may assess a different aspect of animal temperament. This is supported by previous studies which have found behavioural responses to tests primarily involving novel objects are not necessarily related to those primarily involving human interaction [31,32]. In each case we expect a third phase would be a useful addition to the protocol. By using a progression in intensity of novel stimuli in the acclimation protocol, we expected the confidence of the heifers in dealing with each new challenge to gradually develop, however it could be interesting to further examine the influence of each phase of testing on the subsequent phases of testing and determine whether conducting any of the phases independently might provide the same outcomes. Overall, with further refinements and automation using Radio-Frequency Identification (RFID) technology to record Movement Order, we believe this protocol could be a useful and practical tool for assessing the acclimation rate of cattle at a group level.

Contrary to our hypothesis, the rate of acclimation for individual heifers, as measured by changes in times and orders of animals between test days 1–6, did not appear to have any consistent relationships with FT, weight change during and after weaning or individual movement through the acclimation test on day 9. These results may indicate rate of acclimation is not associated with the temperament or production variables measured in this study, however it is more likely that the testing protocol was not appropriate for assessing acclimation rate of animals at an individual level. Although testing animals as a group allows for a more practical test with greater potential commercial applications, it can limit our ability to truly assess the traits of individuals, as an individual’s behaviour can be influenced directly or indirectly by other members of the herd [33]. The method presented in the current paper relies on the premise that animals which acclimate to a new environment faster will move past a novel object quicker than those which acclimate at a slower rate. However in the current test design, the time it takes an animal to move past the object is confounded by the time it takes the animal to reach the object, which is limited by its position in the herd. Further, cattle towards the back of the herd may not have seen the newly added object on day 4 of testing until they had reached the mouth of the funnel, at which point they may have had a limited ability to turn around due to pressure from the animals behind. We suggest a better measure of individual acclimation rate might involve assessing the heifer’s hesitation after reaching the funnel or traffic cone, however this approach also comes with difficulties, such as defining at which point to begin a measure of ‘hesitation’ for each animal. Alternatively, individual testing would permit more uniform exposure of each animal to the challenge and allow for assessment at an individual level, but this again comes with limitations such as the impact of isolation on animal behaviour and an increase in human interaction.

A range of factors may have contributed to the order in which cattle moved through the funnel structure during acclimation rate testing, but we hypothesise that temperament and feeding motivation may have been the most important factors. Temperament describes the behavioural responses of an animal which are consistent over time in a particular context [34]. Flight time is often used as an indicator of temperament in cattle, however it is important to note that there are many aspects or dimensions of temperament which can be represented by a range of other behavioural responses in different contexts [35]. Interestingly, flight time was not related to the First and Last movement groups identified during this study, which likely indicates the acclimation protocol reflected a different aspect of animal temperament than the flight time measure. This is supported by previous studies which found flight time was not related to responses to novel objects [32] and is not impacted by pharmacological manipulation of fear and anxiety [36]. Differences in temperament between the First and Last Movement Groups may have contributed to the variation in growth rate observed post weaning, as temperament is known to relate favourably to growth rate, feed intake and feed efficiency in beef cattle [5,18,19,37,38,39,40,41,42,43,44,45]. It should be noted that many of these studies employed flight time for temperament assessment but that they all used at least one other behavioural measure in conjunction with flight time, such as agitation scores in a crush or small yard.

Feeding motivation is likely to be another important factor contributing to movement order through the funnel. Order of movement on day 1 was not correlated with order on days 2–6. We expect this is because cattle had not yet made the association between the testing procedure and feeding on day 1. From day 2 onwards this association was stronger and animals with increased motivation to feed would have been able to better position themselves within the holding yard to quickly move into the feeding yard once testing began. Furthermore, the protocol is comparable to existing measures of feeding motivation, which typically ask the animal to pay a ‘price’, such as a reduction of normal flight distance seen in this study, to maintain access to feed [46,47,48,49]. Additionally, it is important to consider the way in which temperament and feeding motivation might interact to determine movement order. For instance, an animal which moved through the funnel last may have had a calmer temperament but was not motivated to feed, or it may have been motivated to feed but too nervous to move through the funnel. Further studies utilising additional tests to examine temperament, feeding motivation and a range of other behavioural and physiological variables such as social affiliations within the herd may help to better understand the degree to which each of these factors may have influenced order of movement through the funnel in the current study [50,51].

The variable measured with the most consistent relationship to flight time was the order in which heifers were tested on day 9. That is, the order in which individual heifers were moved out of a holding yard by a human handler prior to testing. Correlations indicated a favourable relationship between FT and order of testing, where animals considered to be calmer according to FT measures were first to be moved out of the holding yard. We expect this may be because more nervous animals were more likely to move to the back of the yard away from the human handler, while less flighty animals remained at, or were pushed towards, the front of the herd closest to the human handler. This result is in agreement with previous studies [37,52], but contrasts with Petherick et al. [53], who found flight time related to general activity in the presence of a human handler but not to proximity or flight distance from the human handler. Interestingly, order of retrieval from the holding yard was not related to the time it took individual heifers to move past a stationary human during acclimation testing on day 9, even though both situations involved human interaction. This reinforces the importance of context when assessing the behavioural responses of an animal to a particular stimulus. The lack of relationship between retrieval order and movement past a stationary human could be due to a number of reasons. Firstly, cattle behave differently to humans which take an active role, such as the handler moving cattle out of the holding yard in the current study, versus a passive role, such as the stationary human ‘obstacle’ at the mouth of the funnel structure [54]. Secondly, heifers were isolated during testing on day 9 which may have impacted on their responses [55]. Finally, movement through the funnel had been strongly associated with feeding while order of retrieval from the holding yard had not been and therefore the time taken to move through the funnel may have been related to feeding motivation. Overall, these findings suggest the order in which cattle are moved through handling facilities may be related to some aspect of animal temperament and could potentially be used as a proxy measure of temperament that is easy to record and could be readily automated. Additionally, these findings demonstrate the need to consider the context in which stimuli are presented and not just the types of stimuli presented when interpreting animal behaviour, which would be important if adapting the test protocol for use on individual animals rather than in a group setting.

## 5. Conclusions

The current study presented a novel protocol designed to measure rate of acclimation in cattle which could be readily automated using RFID technology in commercial settings. While we maintain our hypothesis that a faster ability to acclimate is a desirable phenotype, the protocol presented here was not appropriate for measuring acclimation rate in individual cattle. Nonetheless, we expect the current method could potentially be useful as a measure of acclimation rate at a group level. Further work is required to confirm the degree to which movement order is influenced by temperament, feeding motivation or other factors and to determine any associations it may have with future performance of animals. This study also demonstrated a relationship between order of movement by a human handler and temperament, where calmer animals were quicker to be moved out individually from the rest of the herd.

## Figures and Tables

**Table 1:** Timeline for the acclimation trial indicating when each procedure was performed on the heifers. Procedures are listed in chronological order for each given day of the trial. The different phases of acclimation rate testing are indicated by different colours (phase 1—yellow, 2—orange, 3—blue, 4—green).

Day	Procedures
**0**	Weaned from dams
	Transported to yards
	Flight Time assessed
	Weighed
	Ear tagged
	Blood sample collected
	Numbers painted on rump
	Walked through testing set-up to feed yard
**1**	Acclimation rate tested: no obstacle
**2**	Acclimation rate tested: no obstacle
**3**	Acclimation rate tested: no obstacle
	Flight time assessed
	Blood sample collected
**4**	Acclimation rate tested: traffic cone
**5**	Acclimation rate tested: traffic cone
**6**	Acclimation rate tested: traffic cone
	Flight time assessed
**7**	Acclimation rate tested: human
**8**	Acclimation rate tested: human
**9**	Acclimation rate tested: human (individual)
	Flight Time assessed
	Weighed
	Returned to pasture
**1 month post wean**	Mustered to yards
	Flight Time assessed
	Weighed
**5 months post wean**	Mustered to yards
	Weighed
**10 months post wean**	Mustered to yards
	Weighed

**Table 2:** Spearman’s rank correlation coefficients for Order of movement through the funnel on days 1–6 of acclimation testing. Significant correlations are denoted with bold font and the symbol ‘*’ (*p* < 0.05).

	Day 1	Day 2	Day 3	Day 4	Day 5	Day 6
	Phase 1: no obstruction			Phase 2: traffic cone		
Day 1	-					
Day 2	−0.01	-				
Day 3	0.13	0.46 *	-			
Day 4	0.14	0.28 *	0.59 *	-		
Day 5	−0.02	0.20	0.17	0.38 *	-	
Day 6	0.22	0.22	0.31	0.37 *	0.50 *	-

**Table 3:** Mean weights ± s.e.m. of heifers in the First and Last Movement Groups during and post-weaning. A linear model fitting Movement Group, birthweight and age was used to compare group means between First and Last on Day 0. Linear models fitting Movement Group and Day 0 weight were used to compare the group means at all other time points. Heifers were retrospectively grouped as First or Last depending on whether they were consistently one of the first or last 20 animals to move through the funnel during days 2–6 of acclimation testing.

Time Point	First Movement Group Mean Weight (kg)	Last Movement Group Mean Weight (kg)	t Value	*df*	*p* Value
Day 0	279.4 ± 7.1	272.4 ± 5.9	−0.80	36	0.43
Day 9	269.6 ± 6.6	259.5 ± 6.1	−1.23	35	0.23
1 month post	292.8 ± 7.2	280.2 ± 6.4	−1.60	35	0.12
5 months post	345.0 ± 9.2	311.4 ± 8.3	−3.45	35	0.001
10 months post	518.6 ± 10.2	483.6 ± 7.9	−3.73	35	<0.001

**Table 4:** Pearson’s correlations between order in which cattle were tested on day 9 and mean Flight Time (FT) on days 0, 3, 6, 9 and one month post weaning.

Statistic	Day 0	Day 3	Day 6	Day 9	Post-Wean
r	−0.34	−0.31	−0.48	−0.52	−0.29
*p*	0.016	0.030	<0.001	<0.001	0.039

## Supplementary Materials

The following are available online at http://www.mdpi.com/2076-2615/8/4/51/s1.
